# Detection and allergen analysis of serum IgE in pediatric patients with chronic urticaria

**DOI:** 10.12669/pjms.342.14681

**Published:** 2018

**Authors:** Yi Zhou, Minmin Sheng, Mingyan Chen

**Affiliations:** 1 Yi Zhou, Department of Dermatology, The First People’s Hospital of Changzhou, No. 185, Juqian Street, Changzhou 213003, Jiangsu Province, P. R. China; 2 Minmin Sheng, Department of Dermatology, The First People’s Hospital of Changzhou, No. 185, Juqian Street, Changzhou 213003, Jiangsu Province, P. R. China; 3 Mingyan Chen, Department of Dermatology, The First People’s Hospital of Changzhou, No. 185, Juqian Street, Changzhou 213003, Jiangsu Province, P. R. China

**Keywords:** Allergen, Chronic urticaria, IgE, Serum Skin disease

## Abstract

**Objective:**

To detect the serum IgE and allergen-specific IgE levels of pediatric patients with chronic urticaria, and to analyze the distribution characteristics of allergens.

**Methods:**

Ninety-six patients with chronic urticaria admitted in our hospital, which were not administered antihistamine 10 days before detection or glucocorticoid 20 days before detection, were selected. Their serum IgE levels were measured and 34 antigens were analyzed.

**Results:**

Ninety-two of the ninety-six patients were detected as serum IgE positive (positive rate: 95.83%). The positive serum IgE levels did not significantly change along with season (P>0.05). The positive detection rate of antigens was 95.83% (92/96), and the top five potent antigens included house dust mite, flour mite, histamine, egg yolk and egg white.

**Conclusion:**

Dust mite, as the most common antigen for pediatric patients with chronic urticaria, is prone to variations of specific IgE positive rate along with season. The results may be associated with the persistent warm and humid climate in this region.

## INTRODUCTION

Chronic urticaria is a common, refractory pediatric skin disease with complex pathogenesis. Although conventional drugs can temporarily control the symptoms, radical treatment is difficult and recurrence easily occurs. Therefore, it is crucial to identify allergens and to propose targeted desensitization therapy. Chronic urticaria usually cannot be cured due to complicated etiology, which is caused by Type I or III allergic and non-allergic reactions after allergen contacts. Therefore, exploring pathogenesis and determining allergens are of great significance to the treatment of urticaria.^1^-^3^ Due to different geographical environments, climates and dietary habits, there are large regional differences among the allergens that induce chronic urticaria. The pathogenesis for pediatric chronic urticaria is complex, which has been attributed to infections, allergen stimulation, genetic factors and autoimmune antibodies such as IgE. Although the roles of specific IgEs induced by various allergens in the pathogenesis of atopic dermatitis remain controversial, elevated IgE level has been significantly correlated with atopic dermatitis.^4^-^6^ On the other hand, multiple antigen simultaneous test (MAST) is a quick, sensitive, reliable and specific IgE detection method *in vitro*.^7^

Thereby motived, we herein we aimed to study the positive rates of common allergens in children with type I or type III allergic reactions in this region, the distribution characteristics of various allergens and the relationship with season by MAST.

## METHODS

### Baseline clinical data

Ninety-six patients with chronic urticaria were enrolled in the study. It included 51 boys and 45 girls aged between 4 and 10 years old, (7.2 ± 2.8) on average. The disease courses ranged from 42 to 180 days (93.4 ± 42.7) on average. All patients did not take antihistamines or glucocorticoids 10 d or 20 d before detection. The patients who were in the acute phase of urticaria, or suffered from dermatographism, autoimmune disease or thyroid disease were excluded. MAST was performed.

### Grouping and treatment

The 96 selected children were randomly divided into a treatment group (n=48) and a control group (n=48). The treatment group comprised 26 boys and 22 girls, with the average age of (7.1 ± 2.9) years old and the average disease course of (93.1 ± 43.7) d. The control group consisted of 25 boys and 23 girls, with the average age of (7.3 ± 2.7) years old and the average disease course of (94.0 ± 42.5) d. No statistically significant differences were found in age, gender or duration between the two groups.

In the treatment group, allergen biological waves were reversed and amplified through corresponding programs according to the results of allergen detection and electromagnetic waves, and then input into the body for targeted desensitization therapy which was performed twice a week (about 30 minutes each time) for four consecutive weeks. The control group was given cetirizine hydrochloride tablets orally, 10 mg (once daily) for children over the age of 8 and 5 mg (once daily). The treatment course was four weeks.

A specific allergen IgE kit (BD, USA), which can detect serum total IgE and 34 kinds of specific allergens once, was used. Negativity was determined when 30 IU/mL< IgE level< 90 IU/mL, and positivity was determined when IgE level ≤30 IU/mL or ≥90 IU/mL. The samples were treated according to the kit’s instructions, and then the results were read with a CLA-1 chemiluminescent analyzer (Beijing Sage Creation Science Co., Ltd., China). The children did not take corticosteroids 20 d before blood collection.

This study was approved by the ethics committee of our hospital. Written consent was been obtained before examination and treatment for enrolled patients according to the Declaration of Helsinki and relevant laws in China. All treatments were performed based on the patients’ best interests.

### Evaluation criteria for therapeutic effects

Therapeutic effects were evaluated based on the symptoms and signs before and after treatment.^8^

*Scoring for pruritus:* 0 point, without pruritus; 1 point, mild pruritus; 2 points, moderate pruritus; 3 points, severe pruritus.

*Scoring for reddening:* 0 point, without reddening; 1 point, diameter of reddening <1.5 cm; 2 points, diameter of reddening ranged from 1.5 to 2.5 cm; 3 points, diameter of reddening >2.5 cm.

*Scoring for skin wheal:* 0 point, without skin wheal; 1 point, 1~6 skin wheals; 2 points, 7~12 skin wheals; 3 points: >12 skin wheals.

Efficacy index (EI) = (score before treatment – score after treatment)/score before treatment × 100%. Cured: EI>90%; effective: EI ranged from 70% to 90%; improved: EI ranged from 30% to 69%; ineffective: EI<30%. The percentage of cured and effective cases was used as the total effective rate.

### Statistical analysis

All data were analyzed by SPSS 12.0. Ranked data were compared by rank sum test. P<0.05 was considered statistically significant.

## RESULTS

### Total serum IgE levels in different seasons

The total serum IgE of 92 cases was detected positive in the 96 children with chronic urticaria, with the positive rate of 95.83%. The positive rates of total serum IgE in different seasons were similar (P>0.05) (Table-I).

**Table-I:** Positive rates of total serum IgE levels in different seasons.

Quarter	Case No.	Positive rate (%)
1st	24	25.00%
2nd	21	21.86%
3rd	25	26.04%
4th	22	22.92%

### Allergen detection results

Ninety-two children showed positive results by allergen detection, with the positive rate of 95.83% (92/96). The top five potent allergens were house dust mite, mite, histamine, egg yolk and egg white. Six kinds of allergens were detected at most in one child. The allergen test results are shown in Table-II.

**Table-II:** Main allergens of the 96 cases.

Allergen type	Positive [n(%)]	Allergen type
Dust mites and insects	16 (16.67%)	Flour mite, house dust mite, bee, wasp, hornet
Main allergens	10 (10.42%)	Histamine, egg white, egg yolk, milk, sugar, yeast, latex, rubber, rye
Food additives	8 (8.33%)	Sodium glutamate, potassium glutamate, leucine, octyl gallate, butylated hydroxyanisole
Dairy products	7 (7.29%)	Evaporated milk, buttermilk
Pollens	5 (5.21%)	Dandelion, Dahlia, Chrysanthemum, Fraxinus, Artemisia
Environmental chemicals	3 (3.13%)	Tobacco smoke, amianth, acetone, petroleum, gasoline, waste gas, dimethylbenzene, nitromethane
Fish	3 (3.13%)	Prawn, lobster, crayfish, sea eel, mud eel, trout, sardine
Sweeteners	3 (3.13%)	Honey, cane sugar, sorbitol, xylitol, chocolate cyclohexylammonium salt
Moulds	2 (2.08%)	Mould, Monilia albican, Microsporum canis, Neurospora sitophila, Aspergillus niger
Oils/vinegar	2 (2.08%)	Peanut oil, olive oil, sunflower seed oil, sesame oil
Wood/plant fibers	2 (2.08%)	Walnut, Fraxinus, maple, Spruce, cotton
Edible fungi	2 (2.08%)	Mushroom, chantarelle, toadstool, truffle, bolete
Grains	2 (2.08%)	Wheat bran, barley, naked barley, white flour, corn
Creams	2 (2.08%)	Cream, margarine, butter, oleomargarine
Bean products	2 (2.08%)	Sauces, soy, soybean, bean curd, soy milk
Beverages	2 (2.08%)	Lemon balm leaves, white wine, whiskey, wild cherry wine
Furs	2 (2.08%)	Cat, dog, sheep
Fruits	2 (2.08%)	Blackberry, mango, grape, lemon
Vegetables	2 (2.08%)	Brussels sprout, eggplant
Spices and medicinal herbs	1 (1.04%)	Pepper, aniseed
Mixture	1 (1.04%)	Cyclohexylammonium salt, taurine
Heavy metals	1 (1.04%)	Cd, Pb, Hg
Parasites	1 (1.04%)	Ascaris, pinworm
Clothing and dyes	1 (1.04%)	Nylon, polyamide fiber, thioacetazone, disperse orange
Meats	1 (1.04%)	Beef
Nuts	1 (1.04%)	Walnut
Insecticides	1 (1.04%)	Sulfur
Bacteria	1 (1.04%)	Pertussis
Light allergens	1 (1.04%)	Tetrachloro salicylic aniline
Cosmetics and skin care products	1 (1.04%)	Benzyl alcohol
Diluents	1 (1.04%)	Paraffin oil
Vaccines	1 (1.04%)	Pertussis
Drugs	1 (1.04%)	Ribavirin
Protective agents and detergents	1 (1.04%)	Detergents

### Specific IgE detection rates of allergens in different seasons

As shown in Table-III, the positive rates of specific IgE detection for allergens in different seasons are not significantly different (P>0.05).

**Table-III:** Specific IgE detection rates of allergens in different seasons.

Quarter	Case No.	Positive rate (%)
1st	4	4.17%
2nd	3	3.13%
3rd	5	5.21%
4th	4	4.17%

### Treatment results

The cure rate of the treatment group was 64.58% (31/48), and the total effective rate was 95.83% (46/48). The cure rate of the control group was 37.50% (18/48), and the total effective rate was 77.08% (37/48). The curative effect of the treatment group was significantly better than that of the control group (P<0.05).

## DISCUSSION

Chronic urticaria cannot be effectively treated due to long course and undefined reasons, which is commonly treated by antihistamines.^9^-^11^ Hashimoto et al. reported that the combination of mizolastine and tranilast managed to effectively and safely treat chronic urticaria.^12^ Allergen detection protocols, such as allergen skin prick test, antigen solution skin test and immunoblotting, have widely been used.^13^,^14^

Currently, bio-resonance technology based on the physical wave theory has been applied for allergen detection. Each substance has its own unique electromagnetic waves. Resonance occurs when two electromagnetic waves with the same frequency encounter. The spectrum frequency of each substance is unique. Allergy is a biophysical information phenomenon, contacting which human body may produce allergic prints that only retain the bio-resonance wave of the allergen. When the allergen is encountered again, two identical waves are resonant, and then induce common allergic diseases through biophysical pulse. This resonance wave can be recorded by computer, which can identify allergen and weaken allergy prints through wave transfusion, thereby treating allergic diseases.^15^,^16^ Metz et al. conducted allergen detection for 92 cases of chronic urticaria using bio-resonance technology, all with positive results.^17^

IgE-dependent allergic reaction produces IgE antibodies after human body’s initial contacting with an antigen (allergen). Both the total serum IgE and IgE antibodies specific to a certain composite or purified antigen can be measured hitherto.^18^ Detecting serum specific allergen can reflect whether a patient is susceptible to allergy and whether the allergen exists. It has also been reported that chronic urticaria was closely associated with increase of total IgE.^19^

In this study, allergen detection showed that 92 of 96 children showed positive results, with the positive rate of 95.83% (92/96). The top 5 potent allergens were house dust mite, mite, histamine, egg yolk and egg white. Six kinds of allergens were detected at most in one child. Dust mite (*Dermatophagoides farinae*, house dust mite), also known as powder-fed cutaneous acariasis, can be found in poultry feed, warehouse dust and dust in textile mill, also inhabiting in house dusts, carpets and filling-type furniture. House dust mites, with dander off the body as the bait, are most commonly found in bed dusts, using sheets, pillows, quilts, fabric sofa and carpet as breeding grounds. Dust mites are allergens causing allergic disease, and their dander, eggs, carcasses, secretions and excretions have antigenicity that can induce allergic reaction when an allergic patient contacts with it. Demodicid mite prefers wet places with intolerance to heat, coldness and dryness, and humid and hot environment is most suitable for breeding mites. Thus, demodicid mites undergo most exuberant breeding between June and October each year.^20^

In summary, we found that dust mite was the most common antigen for pediatric patients with chronic urticaria. The results in this study, however, failed to show the seasonal variations of dust mite-specific IgE positive rate, which may be related to the warm and humid climate all year around in this region.

### Authors’ Contribution

**YZ** designed this study and significantly revised this manuscript.

**MS & MC** performed this study, analyzed clinical data and drafted this manuscript.

## References

[1] Gecer E, Erdem T (2012). Aeroallergen prick skin test and autologous serum skin test results in patients with chronic urticaria and their comparison. AnnDermatol.

[2] Spring P, Angelillo-Scherrer A, Bigliardi P (2012). Chronic idiopathic urticaria successfully treated by anticoagulant drugs. EurJ Dermatol.

[3] Missaka RF, Penatti HC, Silvares MR, Nogueira CR, Mazeto GM (2012). Autoimmune thyroid disease as a risk factor for angioedema in patients with chronic idiopathic urticaria:a case-control study. Sao Paulo Med J.

[4] Tedeschi A, Asero R, Lorini M, Marzano AV, Cugno M (2012). Serum eotaxin levels in patients with chronic spontaneous urticaria. EurAnn Allergy Clin Immunol.

[5] Nam YH, Kim JH, Jin HJ, Hwang EK, Shin YS, Ye YM (2012). Effects of omalizumab treatment in patients with refractory chronic urticaria. Allergy Asthma Immunol Res.

[6] Kaplan AP (2012). Treatment of chronic spontaneous urticaria. Allergy Asthma Immunol Res.

[7] Persechino S, Annibale B, Caperchi C, Persechino F, Narcisi A, Tammaro A (2012). Chronic idiophatic urticaria and Helicobacter pylori:a specific pattern of gastritis and urticaria remission after Helicobacter pylori eradication. Int J Immunopathol Pharmacol.

[8] Makino T, Takegami Y, Rehman MU, Yoshihisa Y, Ishida W, Toyomoto T (2012). Maintenance of remission with low-dose olopatadine hydrochloride for itch in well-controlled chronic urticaria. Clin Cosmet Investig Dermatol.

[9] Magen E, Mishal J (2012). Possible benefit from treatment of Helicobacter pylori in antihistamine-resistant chronic urticaria. ClinExp Dermatol.

[10] Iqbal K, Bhargava K, Skov PS, Falkencrone S, Grattan CE (2012). A positive serum basophil histamine release assay is a marker for ciclosporin-responsiveness in patients with chronic spontaneous urticaria. Clin Transl Allergy.

[11] de Vos G, Kravvariti E, Collins J, Tavdy A, Nazari R, Hudes G (2012). Increased allergic sensitization to mugwort in chronic urticaria. Dermatology.

[12] Hashimoto T, Kawakami T, Ishii N, Ishii K, Karashima T, Nakama T (2012). Mizoribine treatment for antihistamine-resistant chronic autoimmune urticaria. Dermatol Ther.

[13] Pezeshkpoor F, Farid Hosseini R, Rafatpanah H, Shakerian B, Jabbari F, Zandkarimi MR (2012). Efficacy of Atorvastatin and Antihistamines in Comparison with Antihistamines plus Placebo in the Treatment of Chronic Idiopathic Urticaria:A Controlled Clinical Trial. Iran J Allergy Asthma Immunol.

[14] Irani C, Jammal M, Asmar G, Hajj H, Halaby G (2012). Chronic urticaria and autoimmune thyroiditis. JMed Liban.

[15] Calamita Z, Pela AB, Gamberini M, Baleotti Junior W, Almeida Filho OM (2012). HLA among Brazilian patients with spontaneous chronic urticaria and positive autologous serum skin test. An Bras Dermatol.

[16] Khan S (2012). Chronic urticaria and use of statins. AsiaPac Allergy.

[17] Metz M, Maurer M (2012). Omalizumab in chronic urticaria. CurrOpin Allergy Clin Immunol.

[18] Chakravarty SD, Yee AF, Paget SA (2011). Rituximab successfully treats refractory chronic autoimmune urticaria caused by IgE receptor autoantibodies. J Allergy Clin Immunol.

[19] Bossi F, Frossi B, Radillo O, Cugno M, Tedeschi A, Riboldi P (2012). Mast cells are critically involved in serum-mediated vascular leakage in chronic urticaria beyond high-affinity IgE receptor stimulation. Allergy.

[20] Maurer M, Altrichter S, Bieber T, Biedermann T, Brautigam M, Seyfried S (2011). Efficacy and safety of omalizumab in patients with chronic urticaria who exhibit IgE against thyroperoxidase. J Allergy Clin Immunol.

